# Dietary Habits in Patients with Ischemic Stroke: A Case-Control Study

**DOI:** 10.1371/journal.pone.0114716

**Published:** 2014-12-15

**Authors:** Ana Rodríguez-Campello, Jordi Jiménez-Conde, Ángel Ois, Elisa Cuadrado-Godia, Eva Giralt-Steinhauer, Helmut Schroeder, Gemma Romeral, Mireia Llop, Carolina Soriano-Tárraga, Montserrat Garralda-Anaya, Jaume Roquer

**Affiliations:** 1 Stroke Unit, Department of Neurology, Neurovascular Research Group, IMIM-Hospital del Mar (Institut Hospital del Mar d'Investigacions Mèdiques), Barcelona, Spain; 2 Departament de Medicina, Universitat Autònoma de Barcelona, Barcelona, Spain; 3 DCEXS, Universitat Pompeu Fabra, Barcelona, Spain; 4 Cardiovascular Risk and Nutrition Research Group (CARIN-ULEC), Program of Research in Inflammatory and Cardiovascular Disorders (RICAD), IMIM-Hospital del Mar (Institut Hospital del Mar d'Investigacions Mèdiques), Barcelona, Spain, CIBER Epidemiology and Public Health (CIBERESP), Instituto de Salud Carlos III, Madrid, Spain; National University of Singapore, Singapore

## Abstract

**Background and Aims:**

Diet appears to have some role in stroke development. The objective of our study was to describe the dietary habits in patients admitted with acute ischemic stroke and compare selected dietary components with healthy controls. Adherence to healthy diet behaviors was also assessed.

**Methods:**

A case-control study of consecutive patients with acute ischemic stroke admitted to the Neurology Department of Hospital del Mar from 2007 to 2010. Patients were matched by age and sex with control subjects. A previously validated nutritional survey was administered to patients and controls. Demographic data, vascular risk factors, caloric intake and dietary nutrients were evaluated. Intention to follow a healthy diet was also assessed in both groups.

**Results:**

A total of 300 acute ischemic stroke patients and 300 controls with evaluation of dietary habits. No differences were observed in vascular risk factors, except smoking habit, diabetes and ischemic heart disease. Stroke patients reported a higher caloric intake: 2444.8(1736.8–3244.5) vs 2208.7(1753.1–2860.7) Kcal, p = 0.001. After adjusting for energy intake, patients had higher intake of proteins (p<0.001; OR 1.02), total cholesterol (p = 0.001; OR 1.04), and breaded foods (p = 0.001; OR 1.94) and lower consumption of probiotic yogurt (p = 0.002; OR 0.88). Compared to patients, control participants indicated greater intention to eat vegetables (p = 0.002; OR 1.5) and whole foods (p = 0.000; OR 2.4) and reduce their intake of salt (p = 0.002; OR 1.7), fat (p = 0.000; OR 3.7) and sweets (p = 0.004; OR 1.7) than patients.

**Conclusion:**

We observed different dietary patterns between stroke patients and controls. Stroke patients have a higher caloric intake and are less concerned about maintaining healthy nutritional habits.

## Introduction

Stroke is one of the leading causes of disability and morbidity in many countries and at all levels of economic development. Lifestyle factors (diet, physical activity, smoking, drinking, and socioeconomic status) are important targets for primary prevention of all cardiovascular diseases, including stroke; cardiovascular risk factors, mainly hypertension, are key to secondary prevention efforts [1].

A relationship between diet and stroke has been previously demonstrated. Diet may influence stroke risk via several mechanisms, but the optimal dietary habits for stroke prevention are not clearly established [2]. In our setting it seems that one of the best dietary patterns for stroke prevention is the Mediterranean diet [3]. The high complexity of nutritional studies makes it difficult to confer a pathophysiological role to isolated dietary components [4]. Complex interactions between the different components of diet may exist, and any effects cannot be attributed to an individual component of the diet but rather to a combination of dietary factors [5]. After a meta-analysis of literature published between 1979 and 2004 assessed optimal dietary habits for stroke prevention, recommendations were made to reduce stroke risk [6]. These include reduced sodium intake and increased consumption of fruits and vegetables, whole grains, cereal fiber and fatty fish. A prudent pattern or a traditional Mediterranean diet, with high intake of these products, legumes and olive oil might also prevent stroke.

Healthy behaviors, especially dietary habits and physical activity, recommended for the primary prevention of stroke and hypertension are quite similar [7], [8]. The recommended healthy lifestyle consists of physical activity, increased fruit and vegetable intake, reductions in weight, salt intake, and saturated and total fat intake, and moderation of alcohol consumption. Individuals with many of these health behaviors are reportedly at lower risk of stroke [1], [9].

The objective of our study was to describe the dietary habits in patients admitted with acute ischemic stroke (AIS) and compare selected dietary components with healthy controls. Adherence to healthy diet behaviors was also assessed.

## Materials and Methods

The case-control study design included patients with a diagnosis of first-ever AIS admitted to the Neurology Department of Hospital del Mar from 2007 to 2010. This is the only public hospital serving a population of 300,000 inhabitants of 3 of the 10 city districts of Barcelona, Spain. Ischemic stroke was confirmed by a neurologist and computerized tomography (CT) scan or magnetic resonance imaging (MRI) to exclude other neurological causes. All patients were prospectively included in the BASICMAR database, an ongoing register of patients with AIS at our hospital.

Patients were matched (1∶1) by age and sex with healthy controls from the REGICOR (Registre Gironí del Cor) database. This register includes a randomized representative sample of men and women of the province of Girona, according to the general population census in 1995, 2000 and 2005 [10]. Dietary data about controls were entered about the same time as for the patients. Both populations studied (Barcelona and Girona) are located in the same region of northeast Spain (Catalunya). Several studies have established that these populations are homogeneous in terms of cardiovascular epidemiology, and have similar eating habits that include a prevalent intake of Mediterranean diet components [11].

### Variables analyzed

Vascular risk factors were recorded following definitions recommended by international consensus: age, sex, weight, body mass index (BMI), current smoking habit, coronary heart disease (documented history of myocardial infarct and/or angina pectoris), diabetes (documented medical history, use of diabetes medication, glycated hemoglobin >6.5%, or new physician diagnosis during follow-up), hyperlipidemia (documented medical history, use of medication, serum cholesterol concentration >220 mg/dl, or serum triglycerides >150 mg/dl), and arterial hypertension (documented prior medical history, use of medication, or evidence of at least two raised blood pressure measurements recorded on different days out of acute stroke phase, defined as systolic >140 mmHg or diastolic >90 mm Hg). A vascular neurologist classified the strokes according to the stroke subtype, as atherothrombotic, cardioembolic, small vessel occlusion, unusual or undetermined according to the Trial of Org 10172 in Acute Stroke Treatment (TOAST) etiological criteria [12].

### Dietary habits

Dietary habits in stroke patients and healthy controls were assessed using a food frequency questionnaire (SFFQ-Supplementary online material) validated for this population. Food frequency intake estimates of 107 participants were compared to ≥10 unannounced 24 h recalls (reference method) [13].

FFQ assessed eating habits during the previous year and was administered during hospitalization after stroke by qualified staff in Catalan or Spanish, depending on the respondent's preference. It was completed by the patient or by a relative if the effects of stroke (speech disorders, aphasia or memory problems) inhibited the patient's response.

The questionnaire used classifies respondents according to a range of nutrient intake. The list contains 166 items representing typical foods in northeastern Spain. It is divided into food categories: dairy products, cereals, vegetables, legumes, eggs, meat and fish, processed meats, oils and fats, fast food, fruit, nuts, beverages, and canned food. Participants indicate the frequency of consumption of each product (daily, weekly, monthly or rarely/never) and specify quantity, presented as half portions and everyday units for each item. For nutritional calculation, the frequency of consumption is multiplied by the portion consumed for each food. Energy consumption and total nutrient intake are calculated from the FFQ questionnaire using the MediSystem 2000 software [14].One year intra-subject reproducibility of 10 nutrients and 7 foods showed an average correlation coefficient of 0.57 (range: 0.43 to 0.77).

In this study we separately analyzed intake of macronutrients which have previously been associated with the incidence of stroke (carbohydrates, proteins and total lipids), lipids subtypes (total cholesterol and monounsaturated, polyunsaturated and saturated fat), fiber, and yogurt containing active lactobacillus species. Correlation coefficients of carbohydrates, protein, total fat, saturated fat, monounsaturated fat, polyunsaturated fat, cholesterol and fiber were 0.36, 0.30, 0.28, 0.21, 0.12, 0.24, 0.20 and 0.37, respectively.

The effects of different ways of preparing food, including methods that are potentially harmful to patients with ischemic stroke, were also analyzed, including consumption of fried and breaded foods. The results for each nutrient are expressed in weight units. Following the standard methodology of nutritional studies [15], we excluded participants with extremely low or high reported energy intakes (those in the lowest and highest 0.5% of the ratio of energy intake over basal metabolic rate; n = 24).The questionnaire includes several items associated with an intention to improve dietary habits to control vascular risk before the stroke event: to increase fruit and vegetable consumption, to eat whole grain products and to reduce intake of meat, fat, sweets and salt.

### Statistical considerations

Quantitative variables were tested for normality by Kolmogorov-Smirnov test and Shapiro-Wilk tests. Those with non-normal distribution were log-transformed to allow the use of parametric tests. All continuous variables were also categorized in quintiles for a more comprehensive and understandable assessment of the associations [15]. For the univariate analyses comparing the study variables between stroke patients and healthy controls, unpaired t-test and Chi squared test were used for continuous variables and categorical variables, respectively. Multivariate analyses included adjustment for caloric intake, age and sex. For each quintile of nutrient intake, risk of AIS and of each stroke subtype was compared for patients and controls. Assuming an initial two-sided *p* value <0.05 for significance and after the Bonferroni Multiple testing adjustment (analyses for 13 items, all of them evaluated in univariate analysis) significant *p* value was set at *p*<0.004 [16]. All analyses were performed with SPSS (version 13.0).

### Ethics statement

The information used in this study was collected from the prospective BASICMAR register with the approval of our local ethics committee (CEIC-PSMAR). All patients gave their informed consent before their inclusion in the register. The control data were collected from the REGICOR study, and all participants signed an informed consent for secondary use of their data.

## Results

**Figure 1:**
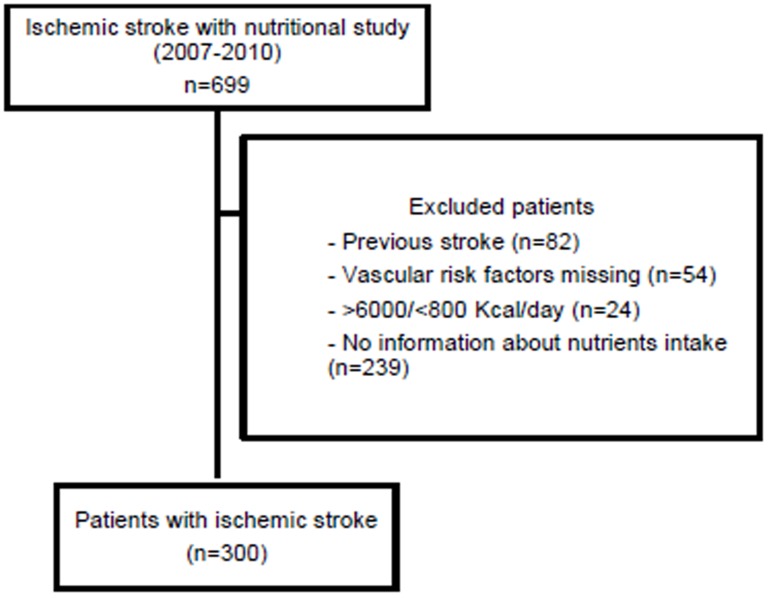
Flow chart of study patients. VRF: Vascular risk factors.

Of 699 ischemic stroke patients admitted between March 1, 2007, and March 31, 2010, complete nutritional analyses were performed in 300 individuals admitted with first AIS (Fig. 1). These patients were matched with 300 healthy controls.

According to the TOAST classification, 48 patients had atherothrombotic etiology (16%), 69 lacunar (23%), 93 cardioembolic (31%), 7 unusual (2.3%) and 83 undetermined (27.7%) due to double pathology or incomplete study.

Mean age of stroke patients was 73.9±12.1 years (range 31–100), similar to controls (73.1±12.1, range 38–86); 53.7% of the patients and 54.1% of healthy controls were male (*p* = 0.75). Regarding vascular risk factors, stroke patients had higher rates of current smoking habit, diabetes and ischemic heart disease, compared to controls. There were no differences in weight, BMI, hypertension or dyslipidemia (Table 1).

**Table 1:** Demographic data and vascular risk factors.

	Stroke patients	Controls	p
	(N = 300)	(N = 300)	
Age, mean±SD	73.9±12.1	73.1±10.8	0.35
Sex (male), n(%)	161(53.7)	162(54.1)	0.75
Weight (Kg)	73.3±13.1	73.3±13.7	0.94
BMI	27.8±4.5	27.7±4.3	0.82
Smoking habit	69(23.5)	46(14.4)	0.004*
Hypertension	206(69.1)	225(63.9)	0.16
Diabetes mellitus	100(34.4)	71(21.8)	0.001*
Dyslipidemia	126(42)	141(43.4)	0.70
Coronary heart disease	41(13.7)	28(8.6)	0.04*

* p<0.05.

SD: Standard deviation.

Kg: Kilograms.

BMI: Body mass index.

We observed a higher caloric intake in stroke patients than in controls: 2444.8(1736.8–3244.5) vs 2208.7(1753.1–2860.7) Kcal, respectively (*p* = 0.001). Macronutrients intake, in absolute values, was higher in AIS patients than in controls. However, in order to assess differences in profiles of nutrition habits, analyses were also weighted by adjusting for global energy intake. After adjusting, AIS patients had higher intake of proteins (*p*<0.001; OR 1.02), total cholesterol (*p* = 0.001; OR 1.04) and breaded foods (*p* = 0.001; OR 1.94) and lower consumption of yogurt with lactobacillus species (*p* = 0.002; OR 0.88) (Table 2). Some differences were observed between stroke subtypes (Table 3): overconsumption of proteins was especially marked for patients with atherothrombotic stroke; higher cholesterol intake was more marked in cardioembolic strokes. There were no dietary differences between lacunar stroke and controls; patients with undetermined stroke differed from controls in their typical food preparation methods.

**Table 2:** Quantitative differences of nutrients in stroke patients and controls, univariate and after adjusting for energy intake.

	Stroke patients	Controls	*p*(r)	*p*(w)	OR(95%CI)
	(N = 300)	(N = 300)			
Energy (Kcal)*	2444.8(1736.8–3244.5)	2208.7(1753.1–2860.7)		0.001‡	
Carbohydrates, g/d†	241.8(181.1–338.5)	228.6(174–298.1)	0.037	0.62	0.99(0.99–1.01)
Proteins, g/d	110.2(81.4–150.4)	97.4(74–121.4)	0.001	0.0001‡	1.02(1.01–1.03)
Total lipids, g/d	109.4(73.3–150.2)	96.8(72.8–133.3)	0.007	0.51	0.99(0.99–1.01)
Total cholesterol, g/d	388.4(270.5–580.1)	320.5(236.6–430.8)	0.001	0.0001‡	1.04(1.02–1.05)
Monounsaturated fat, g/d	52.2(33.6–73.6)	48.6(36.4–6.6)	0.234	0.23	0.97(0.89–1.01)
Polyunsaturated fat, g/d	15.9(10.1–24.9)	14(9.8–19.7)	0.002	0.09	1.02(0.99–1.06)
Saturated fat, g/d	32.4(22.1–46.1)	28.9(19.8–38.6)	0.001	0.2	0.99(0.98–1.01)
Fiber, g/d	27.9(18.8–40.4)	26(18.9–36.2)	0.383	0.83	0.99(0.98–1.01)
Probiotic yogurt	61(21.3)	104(34.3)	0.005	0.002‡	0.88(0.81–0.96)
Fried food	132(44.6)	162(50.8)	0.001	0.19	0.92(0.82–1.04)
Breaded foods	166(56.3)	120(37.9)	0.002	0.001‡	1.94(1.39–2.71)

*p*(r): p-value for the raw analysis, without adjustment for caloric intake.

*p*(w): p-value weighted by adjustment for caloric intake.

OR and *p*(w) are calculated in a weighted model.

*Kcal: Kilocalories. Median(Q1–Q3).

† grams/day.

‡ *p*<0.05.

**Table 3:** Analysis of nutrients by quintile (Q), assessing their association to total acute ischemic strokes (AIS) and each stroke subtype (according to TOAST classification).

	CT vs TAIS	CT vs AT	CT vs LAC	CT vs CE	CT vs Other*
	(n = 300)	(n = 48)	(n = 69)	(n = 93)	(n = 90)
	OR(95% CI)	OR(95% CI)	OR(95% CI)	OR(95% CI)	OR(95% CI)
Carbohydrates					
Q1	1	1	1	1	1
Q2	0.67(0.37–1.21)	1.12(0.34–3.73)	0.88(0.37–2.12)	0.69(0.31–1.54)	1.55(0.66–3.66)
Q3	0.50(0.25–1.00)	1.28(0.38–4.24)	0.41(0.15–1.11)	0.43(0.17–1.08)	0.97(0.38–2.51)
Q4	0.83(0.36–1.88)	1.06(0.27–4.16)	0.28(0.09–0.87)	0.41(0.15–1.18)	0.62(0.20–1.89)
Q5	3.40(1.46–7.92)	1.51(0.30–7.47)	0.24(0.06–0.93)	0.82(0.24–2.76)	1.13(0.38–4.99)
P for trend	0.07	0.96	0.12	0.14	0.16
Proteins					
Q1	1	1	1	1	1
Q2	1.21(0.69–2.11)	0.61(0.16–2.33)	0.86(0.33–2.27)	1.97(0.83–4.69)	1.26(0.51–3.09)
Q3	1.34(1.21–2.49)	1.16(0.32–4.20)	0.49(0.15–1.55)	2.56(0.91–7.19)	1.82(0.69–4.73)
Q4	2.13(1.06–4.27)	2.13(0.52–8.72)	1.61(0.51–5.08)	2.67(0.83–8.57)	2.40(0.82–7.01)
Q5	5.87(2.43–14.21)	10.61(2.01–55.98)	2.58(0.61–11,03)	9.66(2.27–41.08)	5.35(1.41–20.26)
P for trend	0.0001‡	0.001‡	0.05†	0.03†	0.1
Total lipids					
Q1	1	1	1	1	1
Q2	0.55(0.32–0.96)	0.37(0.11–1.21)	0.52(0.19–1.44)	0.61(0.27–1.37)	0.66(0.29–1.49)
Q3	0.66(0.35–1.23)	0.62(0.19–1.98)	0.90(0.32–2.55)	0.58(0.22–1.53)	0.60(0.23–1.52)
Q4	0.58(0.28–1.20)	0.34(0.08–1.39)	0.70(0.21–2.37)	0.76(0.25–2.28)	0.46(0.15–1.37)
Q5	0.04(0.55.0.32)	0.82(0.16–4.28)	1.21(0.27–5.40)	1.00(0.25–3.98)	0.45(0.11–1.79)
P for trend	0.11	0.18	0.46	0.5	0.73
Total cholesterol					
Q1	1	1	1	1	1
Q2	1.39(0.81–2.39)	0.84(0.28–2.53)	1.02(0.38–2.77)	2.39(1.02–5.58)	1.46(0.62–3.45)
Q3	1.41(0.80–2.50)	0.42(0.11–1.57)	0.95(0.34–2.67)	2.07(0.79–5.42)	2.28(0.98–5.31)
Q4	2.81(1.54–5.14)	2.11(0.68–6.54)	2.46(0.88–6.85)	5.24(1.99–13.76)	1.97(0.76–5.15)
Q5	6,46(3.09–13.51)	3.04(0.81–11.32)	5.43(1.66–17.80)	11.93(3.82–37.23)	5.19(1.77–15.26)
P for trend	0.0001‡	0.03†	0.005†	0.0001‡	0.03†
Monounsaturated fat					
Q1	1	1	1	1	1
Q2	0.47(0.27–0.80)	1.36(0.12–1.12)	0.45(0.17–1.22)	0.42(0.19–0.91)	0.68(0.31–1.53)
Q3	0.42(0.23–0.75)	0.41(0.14–1.23)	0.55(1.20–1.49)	0.31(0.12–0.76)	0.53(0.22–1.31)
Q4	0.48(0.25–0.92)	0.39(0.11–1.29)	0.76(0.27–2.17)	0.26(0.09–0.71)	0.67(0.25–1.79)
Q5	0.54(0.24–1.21)	0.23(0.05–1.05)	0.81(0.22–2.96)	0.40(0.12–1.29)	0.79(0.24–2.59)
P for trend	0.02†	0.32	0.47	0.04†	0.64
Polyunsaturated fat					
Q1	1	1	1	1	1
Q2	0.67(0.39–1.15)	0.23(0.06–0.94)	0.94(0.35–2.55)	1.03(0.47–1.03)	0.52(0.24–1.14)
Q3	0.68(0.38–1.21)	0.63(0.20–1.98)	0.95(0.33–2.74)	0.80(0.32–0.80)	0.46(0.19–1.09)
Q4	0.85(0.45–1.61)	0.73(0.21–2.54)	1.31(0.43–4.02)	1.17(0.43–1.17)	0.45(0.17–1.18)
Q5	1.46(0.66–3.22)	1.08(0.23–5.05)	1.93(0.48–7.76)	2.96(0.88–9.91)	0.54(0.15–1.88)
P for trend	0.04†	0.2	0.69	0.1	0.34
Saturated fat					
Q1	1	1	1	1	1
Q2	1.57(0.91–2.72)	0.75(0.26–2.20)	1.65(0.64–4.25)	1.94(0.83–4.52)	1.68(0.71–3.94)
Q3	1.19(0.65–2.18)	0.39(0.11–1.34)	0.70(0.23–2.15)	2.22(0.89–5.57)	1.49(0.58–3.82)
Q4	1.70(0.85–3.41)	0.56(0.16–2.03)	1.15(0.35–3.77)	2.56(0.84–7.80)	2.24(0.78–6.41)
Q5	2.42(1.03–5.66)	1.04(0.24–4.52)	1.78(0.44–7.31)	4.97(1.36–18.13)	1.64(0.43–6.25)
P for trend	0.18	0.31	0.28	0.19	0.54
Fiber					
Q1	1	1	1	1	1
Q2	0.71(0.42–1.20)	0.67(0.22–2.03)	0.52(0.22–1.22)	0.71(0.32–1.58)	1.00(0.46–2.21)
Q3	0.53(0.31–0.92)	0.50(0.15–1.69)	0.59(0.25–1.37)	0.54(0.24–1.20)	0.60(0.26–1.41)
Q4	0.66(0.37–1.16)	1.47(0.51–4.21)	0.47(0.18–1.20)	0.58(0.25–1.34)	1.00(0.40–2.50)
Q5	0.80(0.43–1.48)	1.69(0.54–5.31)	0.34(0.11–1.00)	0.78(0.31–1.91)	1.00(0.40–2.50)
P for trend	0.2	0.17	0.36	0.55	0.52
Probiotic yogurt					
	0.49(0.33–0.72)	0.33(0.14–0.77)	0.41(0.20–0.83)	0.83(0.48–1.44)	0.39(0.21–0.72)
P for trend	0.0001‡	0.01†	0.01†	0.52	0.003‡
Fried food					
	0.73(0.52–1.01)	0.93(0.49–1.77)	0.76(0.24–0.96)	0.99(0.60–1.62)	0.63(0.38–1.04)
P for trend	0.06	0.84	0.009†	0.96	0.07
Breaded foods					
	2(1.43–2.80)	1.77(0.92–3.39)	1.89(1.06–3.34)	1.82(1.09–3.02)	2.53(1.51–4.22)
P for trend	0.0001‡	0.09	0.03†	0.02†	0.0001‡

OR for each quintile (Q) explains the risk of this Q compared to Q1, with a global p-value for the trend. All models were adjusted for caloric intake, age and sex.

CT: Controls, TAIS: Total Acute Ischemic Strokes, AT: Atherothrombotic Strokes, LAC: Lacunar Strokes, CE: Cardioembolic Strokes.

* Other group comprises stroke of undetermined etiology and unusual subtype.

† p<0.05.

‡ p<0.004 (p value after Bonferroni's correction).

Regarding awareness of healthy dietary habits, control participants were more likely to indicate an intention to follow a healthy diet as recommended by stroke prevention guidelines. They reported a higher intention to eat vegetables (*p* = 0.002; OR 1.5) and whole foods (*p* = 0.000; OR 2.4) and to reduce salt intake (*p* = 0.002; OR 1.7), fat (*p* = 0.000; OR 3.7) and sweets (*p* = 0.004; OR 1.7) than did ischemic stroke patients. There was no difference in the intent to increase the consumption of fruit and fish, reduce intake of meat or follow a specific diet (Table 4).

**Table 4:** Intention to follow healthy dietary habits in patients and controls.

	Stroke patients	Controls	P	OR (95%CI)
	(N = 300)	(N = 300)		
Increase consumption of fruit	205(71.9)	245(76.3)	0.22	1.2(0.9–1.8)
Increase consumption of vegetables	206(72.3)	258(79)	0.02*	1.5(1.1–2-2)
Increase consumption of fish	181(63.3)	201(62.6)	0.86	0.9(0.7–1.3)
Reduce consumption of meat	179(63)	214(66.5)	0.37	1.1(0.8–1.6)
Reduce fat intake	204(71.3)	286(90.2)	0.001*	3.7(2.4–5.8)
Reduce sweets intake	197(69.1)	256(79.3)	0.004*	1.7(1.2–2.5)
Intake of whole foods	40(14.1)	89(28.3)	0.001*	2.4(1.6–3.6)
Low salt intake	193(67.5)	254(78.6)	0.002*	1.8(1.2–2.5)
Follow a specific diet	33(11.6)	41(13)	0.60	1.1(0.7–1.8)

* p<0.05.

## Discussion

The present study assessed dietary habits in a series of ischemic stroke patients and healthy controls from a similar population. The differences in diet included higher total caloric, protein, and total cholesterol intake and lower consumption of probiotic yogurt among stroke patients, compared to controls. Stroke patients also ate more breaded foods. Some differences were detected between stroke subtypes, notably the intention to follow health dietary habits, which was greater among controls than in the patient group.

The majority of nutritional studies have been done in coronary heart disease (CHD) patients, and have shown that the Mediterranean diet, rich in alphalinolenic acid, reduces CHD recurrence [17]. Reducing total fat intake also reduces cardiovascular risk, and there is an inverse relationship between consumption of fiber, fruits and vegetables and the development of cardiovascular diseases [18]. In Greece, the ATTICA Study reported that consumption of cereals, fish, hardtack (crackers) and olive oil were related to low risk of cardiovascular diseases, while sweets, red meats, margarine, salted nuts, cured cheeses and alcohol were related to high risk [19].

Regarding stroke, previous cohort studies and a 2006 meta-analysis have reported a reduction of stroke risk with the consumption of fruits, vegetables, cereals, folate, fish, olive oil and Mediterranean diet [2]. In the meta-analysis, data were limited or inconsistent concerning magnesium, calcium, antioxidants, total fat, fats other than fish oil, cholesterol, carbohydrates and animal proteins [6].

An inverse relationship has been reported between consumption of fruits and vegetables and the risk of ischemic stroke [20]. This benefit was confirmed in the case of higher intake of vegetables and white fruits, mainly pears and apples: each increase of 25 g/d in white fruit and vegetable consumption was associated with a 9% lower risk of stroke [21].

Several potential biological mechanisms may explain how overall dietary patterns are related to the risk of CHD and ischemic stroke, among them effects on blood pressure, blood lipid levels, blood homocysteine concentrations, oxidative stress, endothelial function, inflammation, and insulin sensitivity [22]. Experimental data in both animals and humans suggest an association between increased dietary fiber intakes and improved plasma lipid profiles, including reduced low density lipoprotein cholesterol concentrations [23].

We observed that stroke patients consumed more protein than the control group. This is controversial, because previous studies suggest that high protein intake may reduce the risk of stroke. Although one recent study suggests that dietary protein intake is inversely associated with risk of stroke in women [24], in another cohort higher intake of red meat was associated with an elevated risk of stroke in both sexes [25], so it has been suggested that the stroke risk may be reduced by replacing red meat with other dietary sources of protein. However, it seems that epidemiological data on protein intake in relation to stroke risk are limited and inconsistent [26]. This inconsistency could be due to the origin of the proteins (i.e., animals vs vegetables).

In our study, stroke patients consumed significantly more total cholesterol -but not total lipids, saturated, monounsaturated or polyunsaturated fat- as a proportion of their global caloric intake, compared to healthy controls. It is possible that differences in dietary habits and the type of lipids consumed explain the discrepancy with other populations that have been studied. Another possible explanation is the sample size, which was not broad enough to detect small differences.

Regarding fiber consumption, we found no significant differences between stroke and control participants, and did not observe the trends of previous studies that strongly suggest a protective effect of whole grain foods on risk of stroke [27]–[29]. Most of the literature related to fiber intake comes from the USA and Japan, where the subtypes of fiber may be consumed in different proportions than in our Mediterranean population. This would likely explain the differences between population results. Consumption of whole grains improves glucose insulin homeostasis and endothelial function, and possibly reduces inflammation and improves weight loss [2].

Yogurt with active lactobacillus species was the only food item that had a protective effect in our study. Despite the benefits to the human body attributed to bifidus bacteria, this nutrient has not been previously studied in stroke patients; more studies are needed to analyze this association and confirm our result.

Information about the relationship between stroke subtypes and diet is scarce. In our study, we observed some differences between nutritional factors based on stroke subtype. Higher consumption of proteins and cholesterol was observed only in patients with atherothrombotic or cardioembolic stroke, compared to controls; no differences were observed in other stroke subtypes. One previous case-control study (124 ischemic stroke patients and 50 controls, aged <65 years) reported a worse dietary score for cardiovascular protection in atherothrombotic stroke patients [5].

Food-related behavioral counseling to promote a healthy lifestyle for cardiovascular disease prevention is an effective strategy in populations at risk [1]. Current recommendations consist of weight reduction, reduction of salt intake, increase in fruit and vegetable intake, decrease in saturated and total fat intake, physical activity, and moderation of alcohol consumption [8]. Individuals with many of these health behaviors are at lower risk of stroke. In our series, healthy controls were more likely to follow these dietary recommendations, primarily indicated by their intention to consume more vegetables and whole foods, and to reduce their intake of salt, fats and sweets. This could be somewhat paradoxical because stroke patients have more vascular risk factors and therefore we might assume that they have been advised by their family physician to strictly control their vascular risk.

However, the INTERMAP study [30] found that individuals with low cardiovascular risk have higher intake of vegetable protein, fiber, magnesium, non-heme iron, and potassium; lower energy intake; lower intake of cholesterol, saturated fatty acids and animal protein; and lower 24-hour urinary sodium, compared with individuals at higher cardiovascular risk. This corresponds to a higher reported intake of fruits, vegetables, grains, pasta/rice and fish, and lower intakes of meats, processed meats, high-fat dairy and sugar-sweetened beverages. Our study results in stroke patients concur with these food intake results.

With respect to sodium intake, recent studies in stroke patients have demonstrated that dietary recommendations for primary prevention of cardiovascular diseases, including stroke, should include reduced salt intake [31]. In our study, the controls followed this specific recommendation with more interest than stroke patients.

A limitation of the study is the lack of data on alcohol consumption by the control group, so it is not possible to compare the two groups for adherence to the traditional Mediterranean diet. We also did not have information about exercise or socioeconomic status, which could influence the dietary habits of both populations. In some stroke patients who could not answer for themselves, a proxy responded to the FFQ for them. This could compromise the comparability of dietary information between patients and controls and affect confidence in the conclusion.

Another limiting factor could be the age of the patients included. Published studies have postulated that in patients older than 65 years the influence of dietary changes due to aging or the occurrence of other health problems likely affect the energy intake [32]. We analyzed populations separately by age group (older than 65 years vs 65 years and younger), and observed the same differences in demographic data and nutritional habits between stroke patients and controls in both populations. These findings support the consistency of the data regardless of energy intake and nutrients in the two age groups. Finally, the limited sample size could have affected the capacity to identify differences in isolated nutrients or stroke subtypes that might be significant in a larger study.

In summary, we observed clear differences between AIS patients and controls in dietary patterns, and patients were less concerned about maintaining healthy nutritional habits. These data support previous studies indicating that dietary habits have a role in the development of stroke. The present work is the first in the literature that evaluates the relationship between dietary components and lifestyle habits and ischemic stroke in Spain, based on a representative population from a northeastern region of the country. Further studies with larger numbers of participants are needed to confirm these results, although the complexity of dietary analysis makes it difficult to conduct randomized clinical trials for nutritional primary prevention of stroke.

## Supporting Information

S1 Figure **SFFQ: Supplementary food frequency questionnaire.**
(TIF)

S2 Figure **SFFQ: Supplementary food frequency questionnaire.**
(TIF)

S3 Figure **SFFQ: Supplementary food frequency questionnaire.**
(TIF)

S4 Figure **SFFQ: Supplementary food frequency questionnaire.**
(TIF)

S5 Figure **SFFQ: Supplementary food frequency questionnaire.**
(TIF)

S6 Figure **SFFQ: Supplementary food frequency questionnaire.**
(TIF)

S7 Figure **SFFQ: Supplementary food frequency questionnaire.**
(TIF)

S8 Figure **SFFQ: Supplementary food frequency questionnaire.**
(TIF)

S9 Figure **SFFQ: Supplementary food frequency questionnaire.**
(TIF)

S10 Figure **SFFQ: Supplementary food frequency questionnaire.**
(TIF)

S11 Figure **SFFQ: Supplementary food frequency questionnaire.**
(TIF)
