# Supplementary figures and images for: Dietary Habits in Patients with Ischemic Stroke: A Case-Control Study
Source: PLoS One. 2014 Dec 15;9(12):e114716. doi: 10.1371/journal.pone.0114716 (PMC4266513; doi:10.1371/journal.pone.0114716)

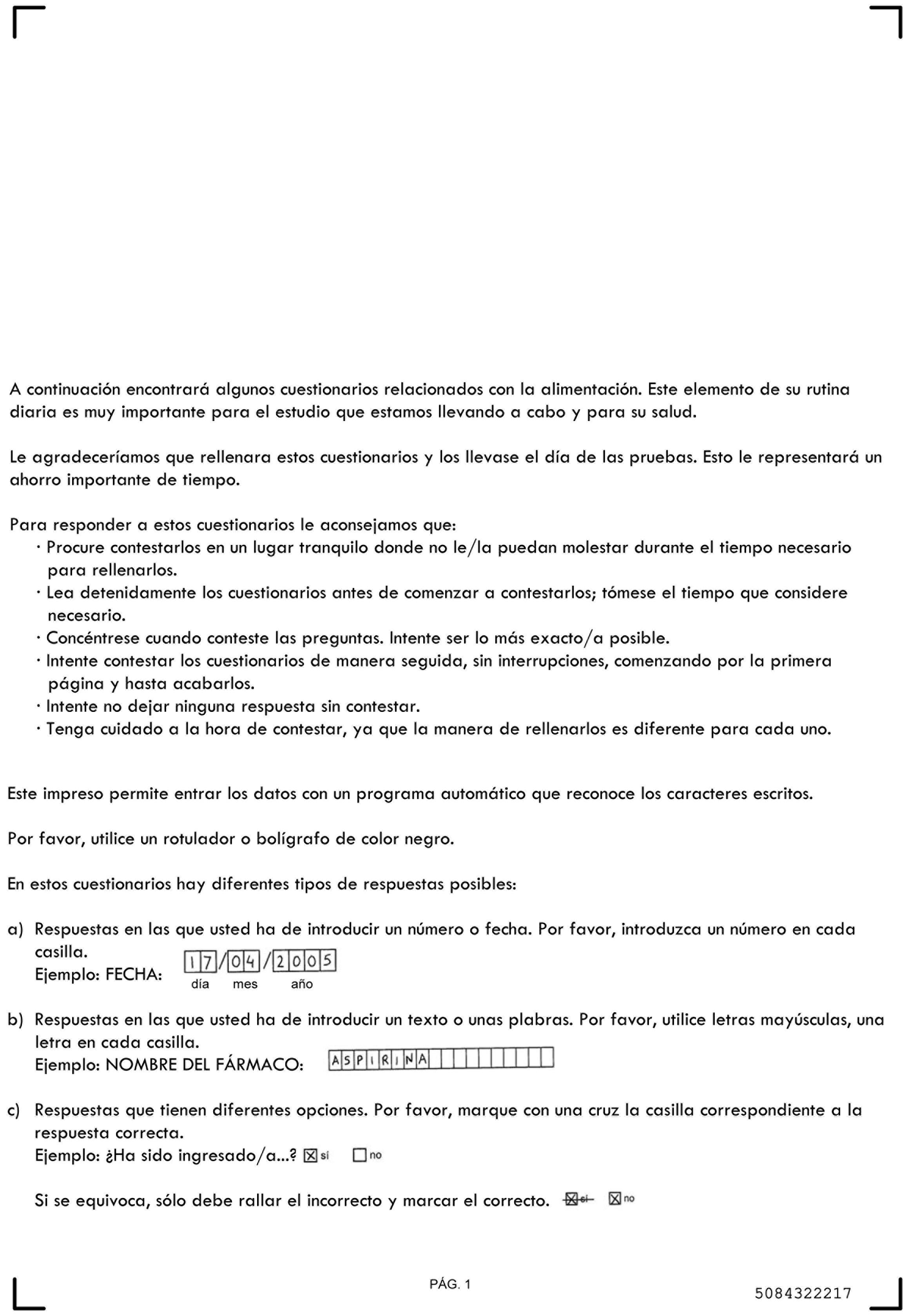

Supplement: S1 Figure — SFFQ: Supplementary food frequency questionnaire. (TIF) [file pone.0114716.s001.tif]

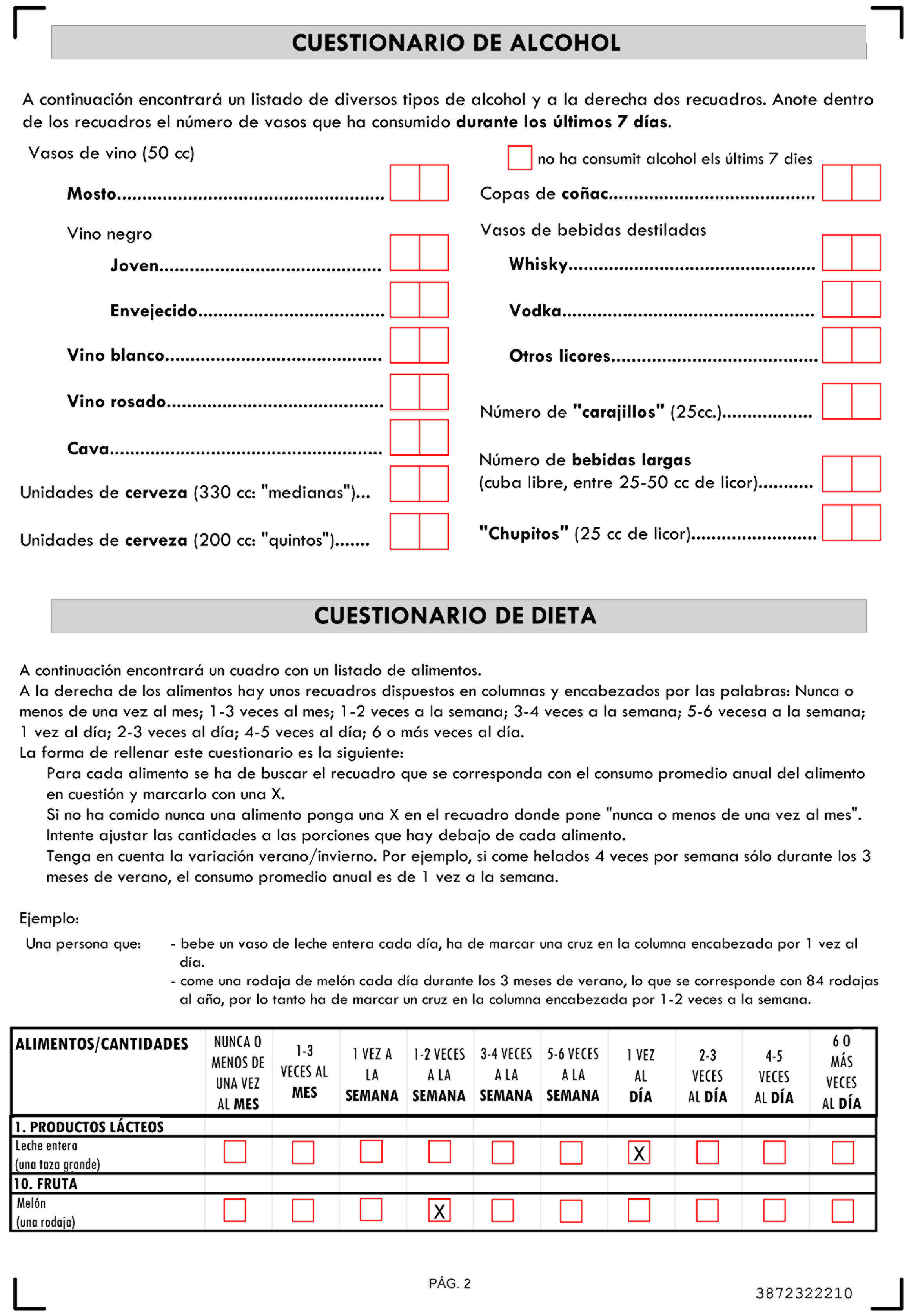

Supplement: S2 Figure — SFFQ: Supplementary food frequency questionnaire. (TIF) [file pone.0114716.s002.tif]

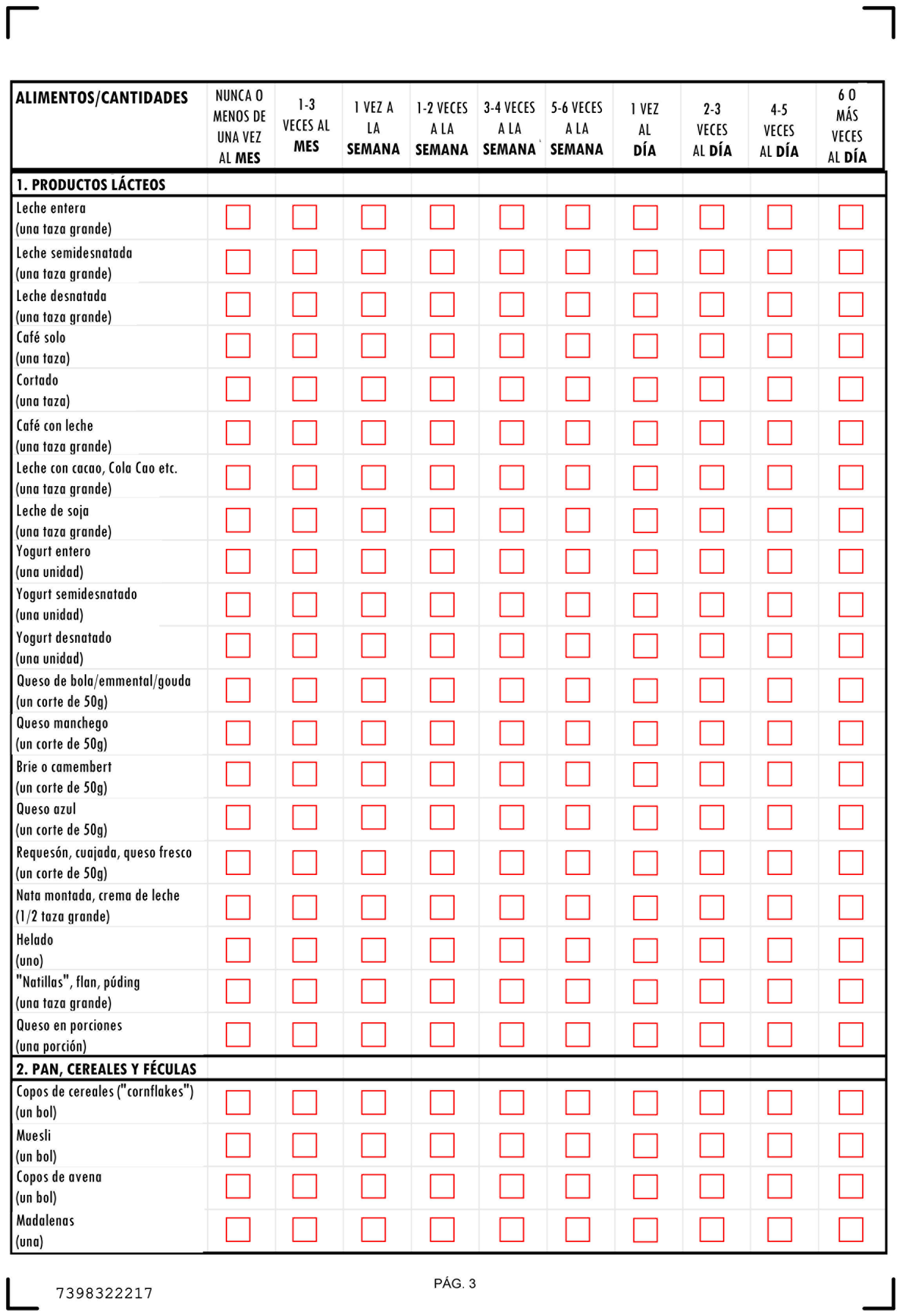

Supplement: S3 Figure — SFFQ: Supplementary food frequency questionnaire. (TIF) [file pone.0114716.s003.tif]

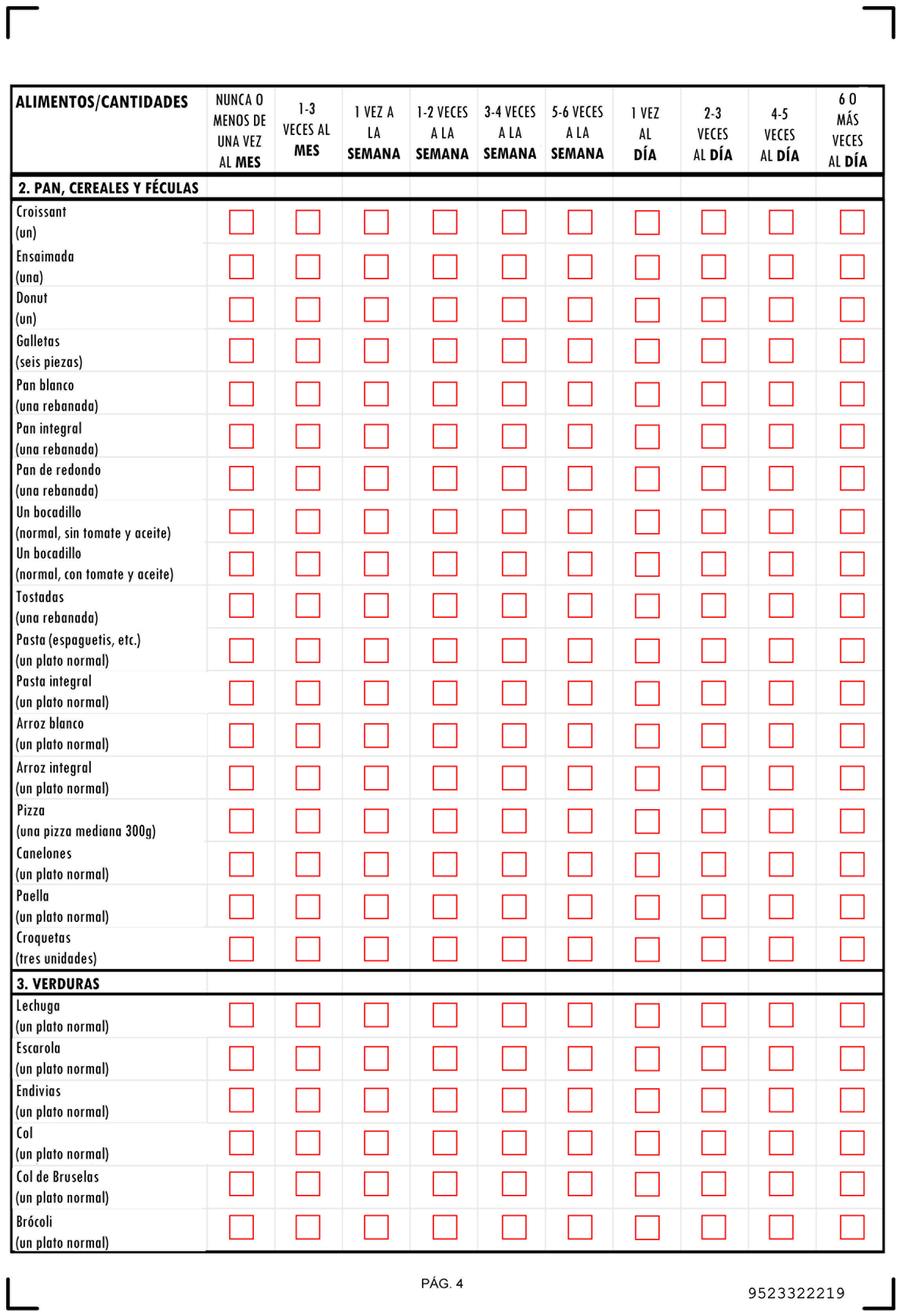

Supplement: S4 Figure — SFFQ: Supplementary food frequency questionnaire. (TIF) [file pone.0114716.s004.tif]

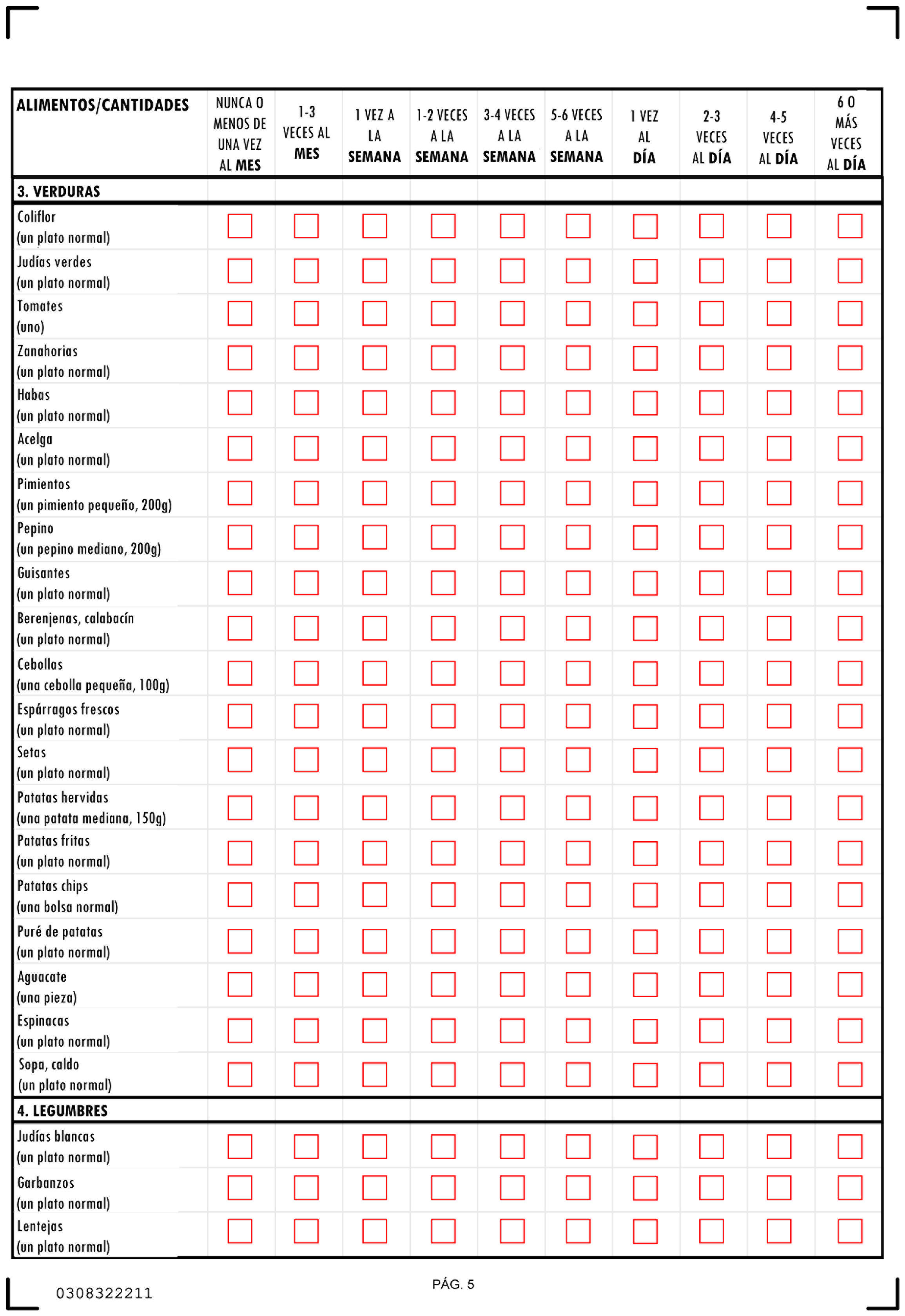

Supplement: S5 Figure — SFFQ: Supplementary food frequency questionnaire. (TIF) [file pone.0114716.s005.tif]

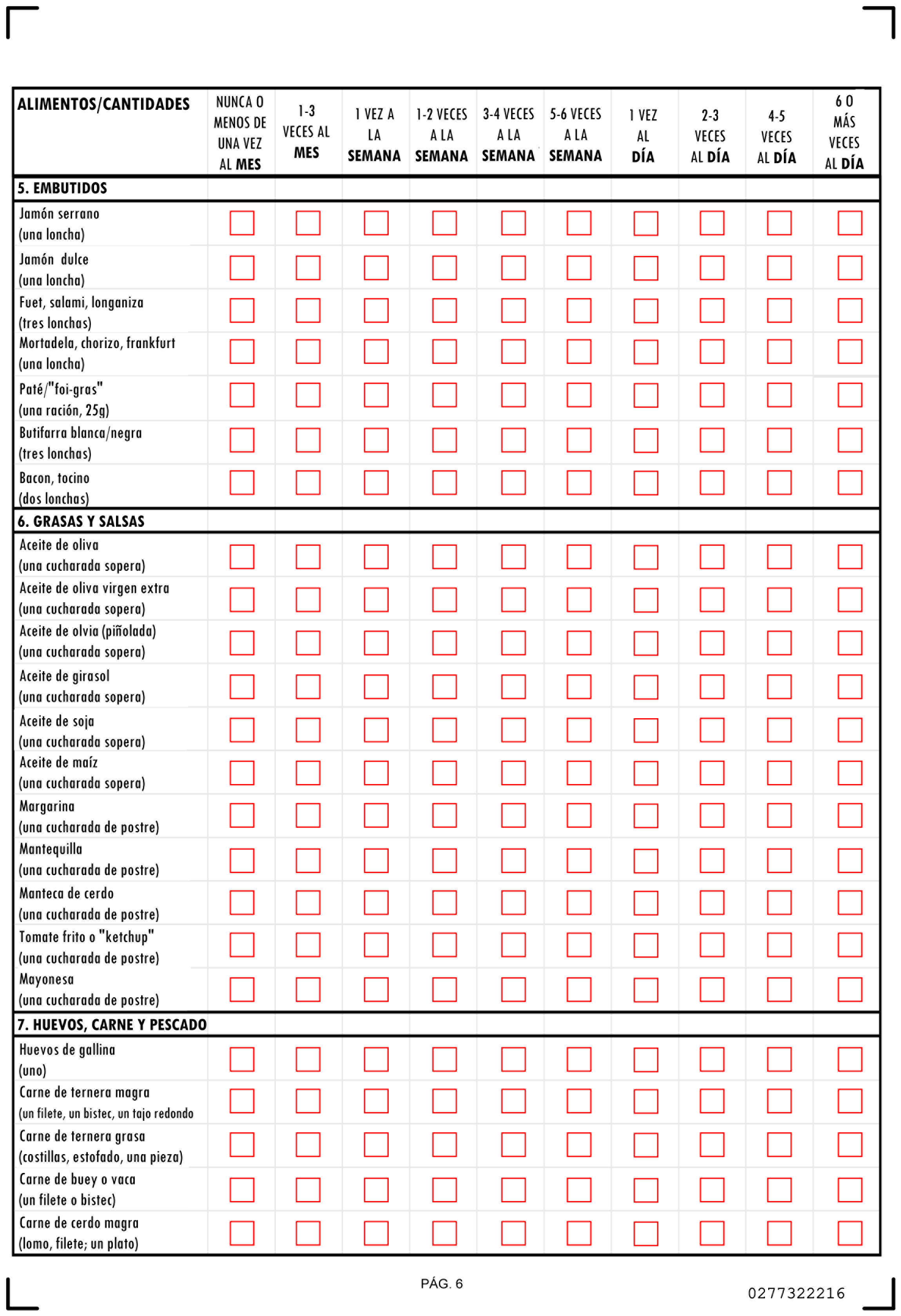

Supplement: S6 Figure — SFFQ: Supplementary food frequency questionnaire. (TIF) [file pone.0114716.s006.tif]

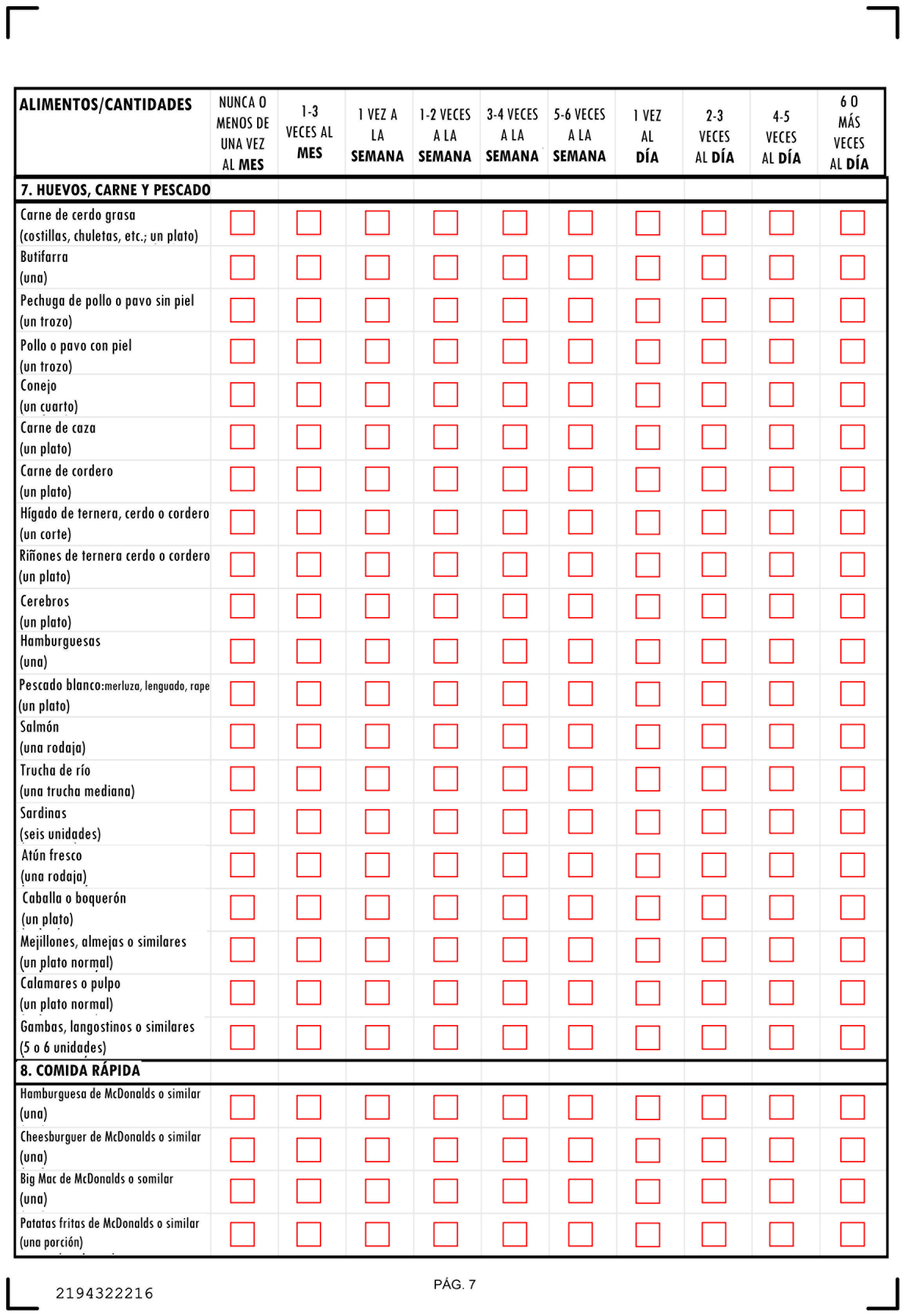

Supplement: S7 Figure — SFFQ: Supplementary food frequency questionnaire. (TIF) [file pone.0114716.s007.tif]

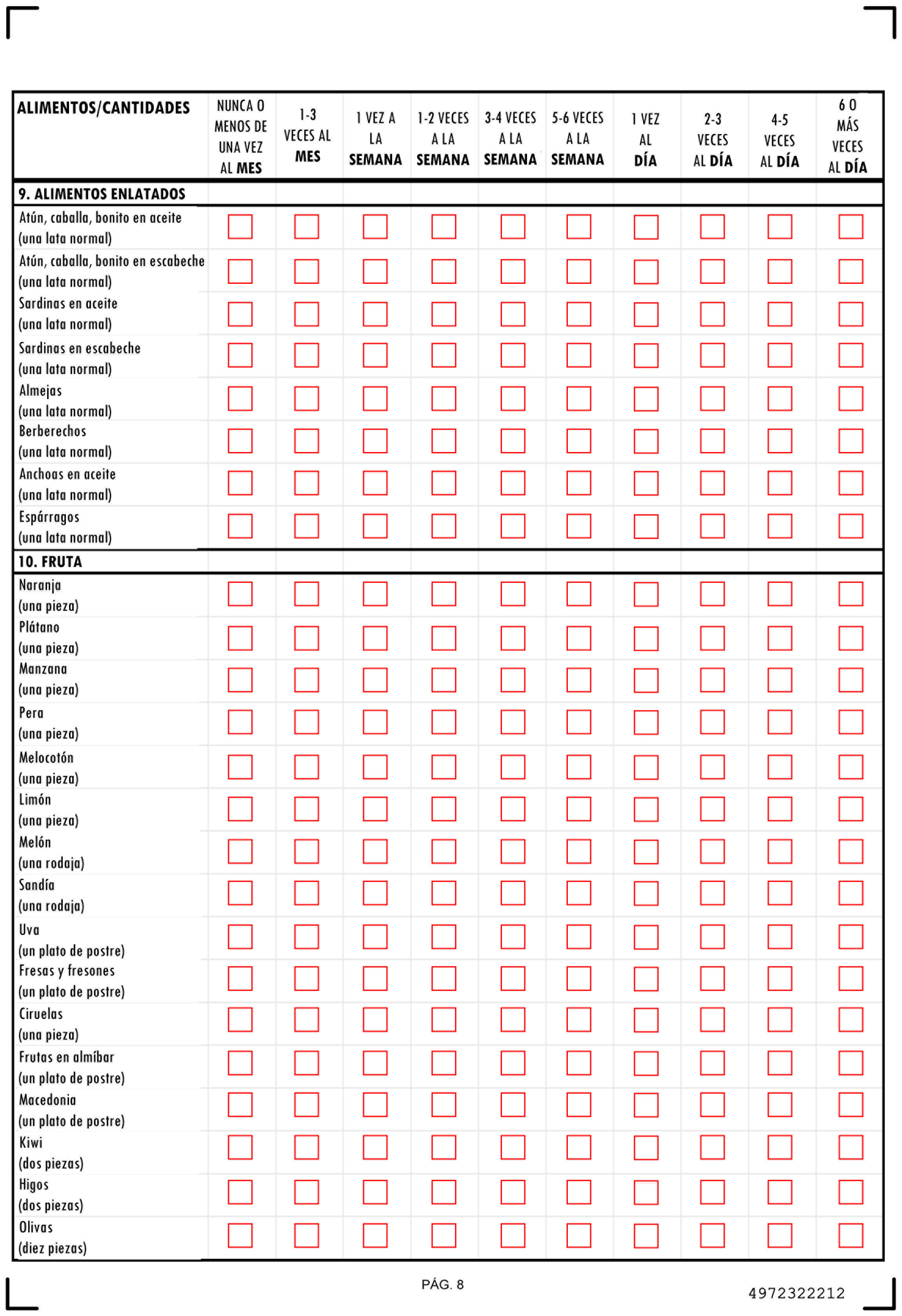

Supplement: S8 Figure — SFFQ: Supplementary food frequency questionnaire. (TIF) [file pone.0114716.s008.tif]

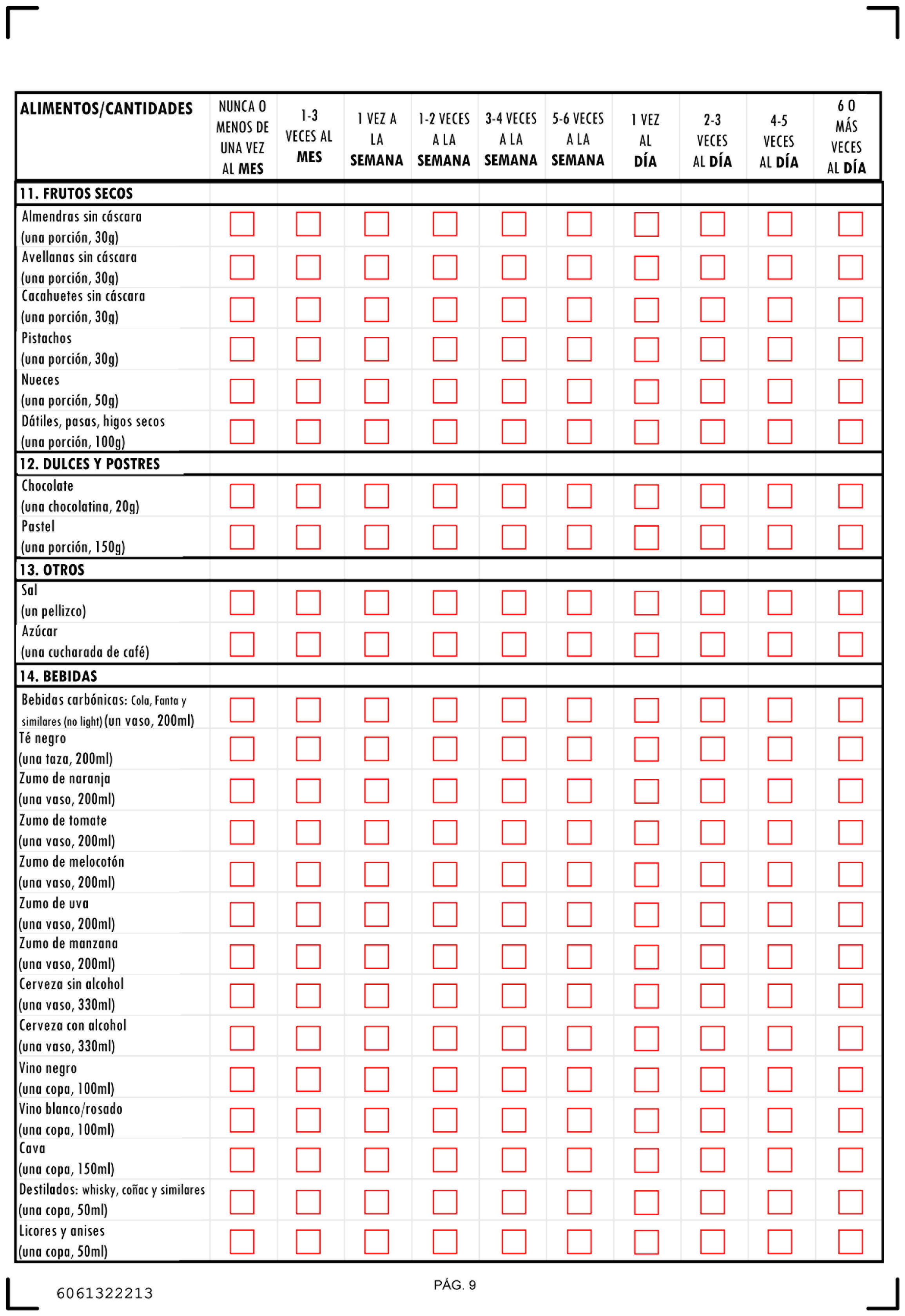

Supplement: S9 Figure — SFFQ: Supplementary food frequency questionnaire. (TIF) [file pone.0114716.s009.tif]

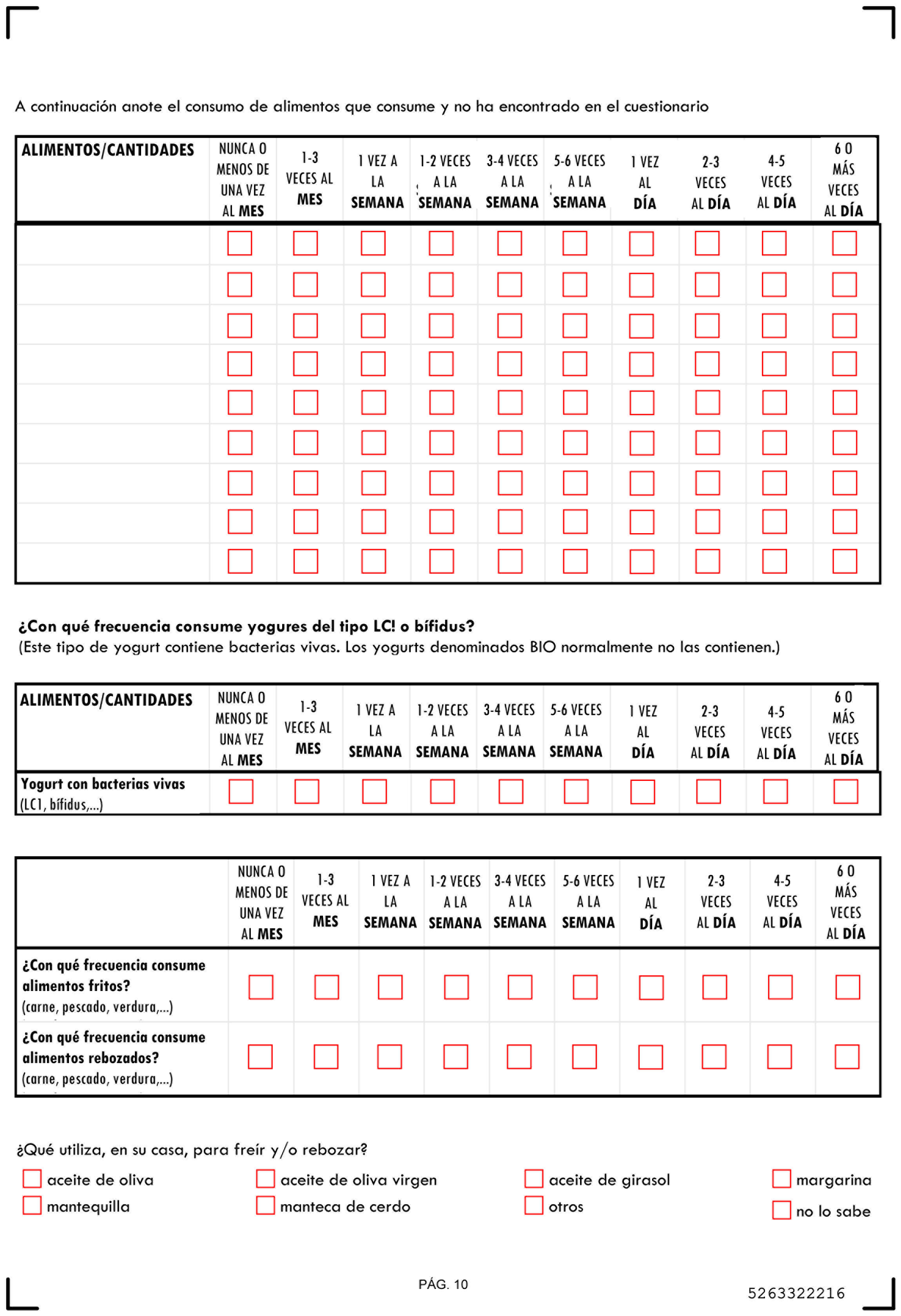

Supplement: S10 Figure — SFFQ: Supplementary food frequency questionnaire. (TIF) [file pone.0114716.s010.tif]

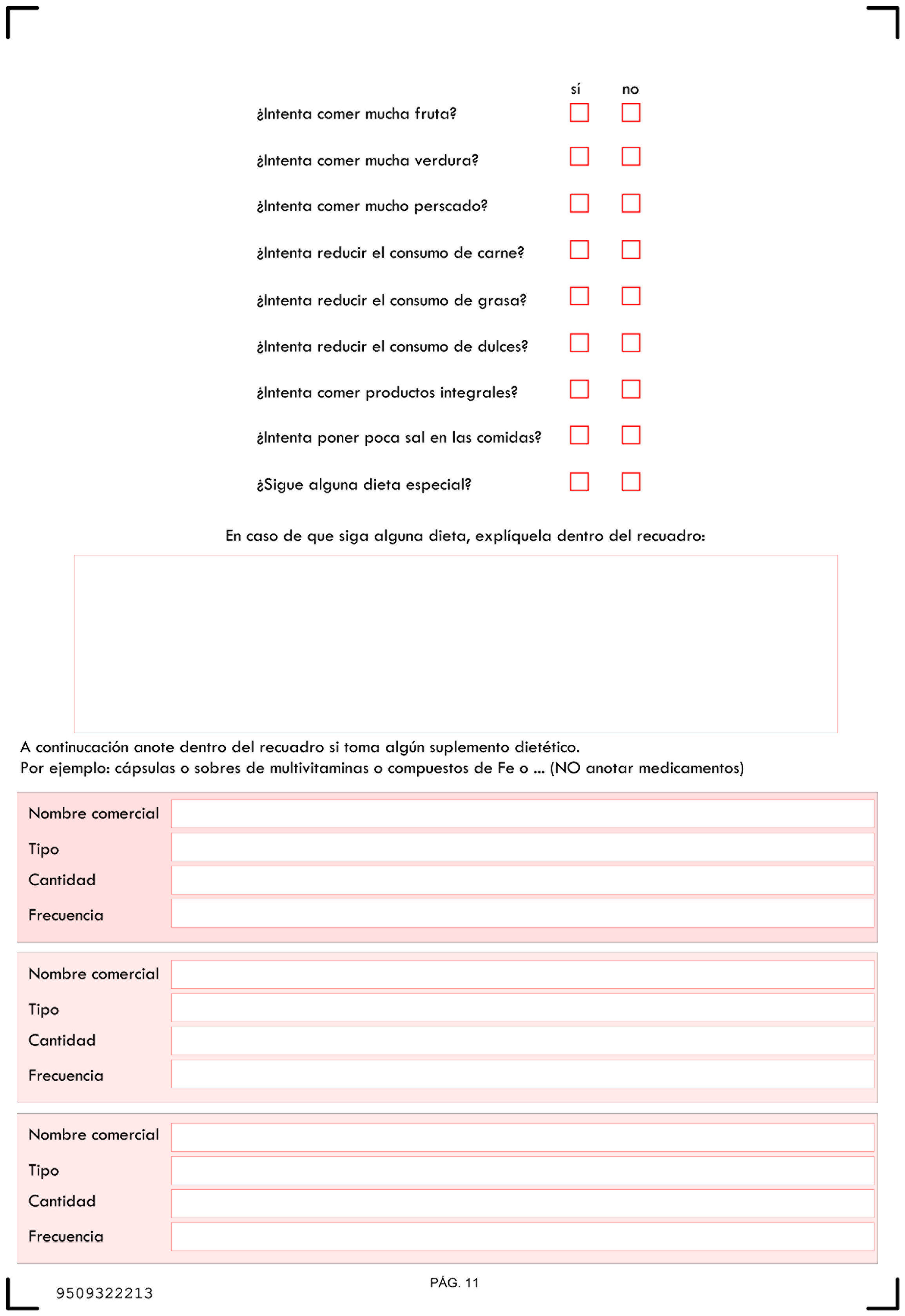

Supplement: S11 Figure — SFFQ: Supplementary food frequency questionnaire. (TIF) [file pone.0114716.s011.tif]
